# A Comprehensive Narrative Review on Autonomic Nervous System Pathophysiology in Spinal Cord Injury and the Use of Invasive and Non-Invasive Electrical Stimulation for Recovery

**DOI:** 10.3390/brainsci16101139

**Published:** 2026-10-21

**Authors:** Karina Yazmín Sánchez-Lastra, Rossy Attie-Harari, María Fernanda Castillo-Cuadros, Carlos A. Cuellar, Adriana Cristina Pliego-Carrillo

**Affiliations:** 1 Facultad de Medicina, Universidad Autónoma del Estado de México, Toluca 50180, Estado de México, Mexico; ksanchezl002@alumno.uaemex.mx; 2 Facultad de Ciencias de la Salud, Universidad Anáhuac México, Huixquilucan 50180, Estado de México, Mexico; rossy.attieha@anahuac.mx (R.A.-H.); 00407562@anahuac.mx (M.F.C.-C.); 3 School of Sport Sciences, Universidad Anáhuac México, Huixquilucan 50180, Estado de México, Mexico

**Keywords:** spinal cord injury, epidural stimulation, transcutaneous spinal cord stimulation, neuromodulation, invasive neuromodulation, non-invasive neuromodulation, autonomic nervous system, autonomic dysfunction, cardiovascular function, respiratory function

## Abstract

**Highlights:**

**What are the main findings?**

- Both eSCS and tSCS demonstrate potential to normalize blood pressure to physiological levels and mitigate orthostatic hypotension, though these cardiovascular gains are largely transient and depend on ongoing active stimulation.
- Targeted neuromodulation enhances respiratory muscle strength and expiratory airflow (improving voluntary cough and airway secretion clearance), while also reducing neurogenic bowel and bladder incontinence, increasing bladder capacity, and restoring sexual/thermoregulatory responses.

**What are the implications of the main findings?**

- Electrical neuromodulation holds potential as a multifaceted therapeutic strategy to restore non-motor functions, thereby improving independence and daily quality of life for individuals with spinal cord injury (SCI).
- Future research must shift from anecdotal or secondary reporting to rigorous, well-powered clinical trials that utilize objective, dedicated biomedical instrumentation alongside standardized outcome scales to systematically optimize stimulation parameters.

**Abstract:**

Spinal cord injury (SCI) significantly reduces both quality of life and life expectancy due to the loss of motor and sensory function, as well as its impact on autonomic dysfunction (AD). Complications related to AD include impaired sphincter control and thermoregulation, orthostatic hypotension, respiratory difficulties, sexual dysfunction, and gastrointestinal complications, among others. These complications limit physical rehabilitation capacities, exacerbating SCI-related challenges and increasing healthcare costs. Currently, no effective clinical treatments exist to restore impaired autonomic neural control. However, invasive and non-invasive neuromodulation strategies such as epidural spinal cord stimulation (eSCS) and transcutaneous spinal cord stimulation (tSCS), respectively, have demonstrated promising results in improving autonomic function. Despite these advances, drawing definitive conclusions remains challenging because studies vary in sample size, stimulation parameters, session numbers, and assessment methods. This narrative review outlines the primary consequences of SCI on the autonomic nervous system (ANS) and evaluates the effects of eSCS and tSCS on cardiovascular, respiratory, gastrointestinal, sphincter control, thermoregulation, and sexual function, while highlighting the diversity of approaches in current studies.

## 1. Introduction

Spinal cord injury (SCI) causes a sudden and profound shift in an individual’s life, provoking irreversible restrictions through partial or complete loss of movement and sensation and disruptions in autonomic nervous system (ANS) function. These impairments significantly affect quality of life and reduce independence [1,2,3]. Each year, between 250,000 and 500,000 people worldwide suffer an SCI, facing a risk of premature death that is two to five times higher than that of the general population, with survival rates being the lowest in low- and middle-income countries [4,5,6]. The severity of motor, sensory, and autonomic disabilities following SCI is closely linked to the level of SCI and the extent of damage to nervous tissue. Additionally, SCI patients frequently suffer from associated complications such as pressure ulcers, urinary tract infections, pain, osteoporosis, and muscle atrophy, among others [7,8,9], with differences between sexes [10]. Despite the extensive autonomic dysfunctions caused by SCI, affecting cardiovascular, respiratory, gastrointestinal, urinary, thermoregulatory, and sexual functions, research on therapeutic strategies has largely prioritized motor function recovery, focusing on restoring movement in the limbs and trunk.

Since the late 1970s, epidural spinal cord stimulation (eSCS) has emerged as an option to treat SCI, primarily to alleviate chronic pain and spasticity [11]. By the late 1990s and throughout the early 2000s, studies began to describe both the immediate and mid-term effects of eSCS on partial motor recovery in individuals with complete SCI [12,13,14]. These findings led to the development of novel research approaches and discoveries in both animal models and human studies [15,16,17]. A few years later, tSCS emerged as a non-invasive strategy that demonstrated the activation of spinal motor circuits as previously described with eSCS [18,19,20,21]. Since then, research has primarily focused on improvements in motor function. However, eSCS and tSCS also mitigate autonomic dysfunction (AD), improving cardiovascular, respiratory, gastrointestinal, urinary, thermoregulatory, and sexual systems.

Although one strategy is invasive and the other non-invasive, their mechanisms of action on the nervous system appear to overlap. In both cases, the applied electric current follows heterogeneous paths according to tissue impedance, depolarizing large-diameter primary afferent fibers of the dorsal roots, which in turn generate monosynaptic and polysynaptic responses [20]. This synaptic activation engages spinal circuits comprising interneurons and motoneurons, thereby modulating autonomic system responses, likely via the somato-autonomic reflex. Furthermore, the propriospinal system appears to mediate this autonomic activation through projections to laminae VII and X, where preganglionic neurons are located [22]. A preclinical study described the activation of sympathetic preganglionic neurons without directly activating the dorsal columns or dorsal horn/intraspinal neurons, confirming the critical role of dorsal root afferent fiber depolarization [23]. Therefore, both neuromodulation modalities are associated with plastic effects, as the effects produced have been observed to persist beyond the acute and medium-term stages and include reorganization from supraspinal centers [24,25]; however, it has not yet been clearly established whether these effects are permanent once a plateau in autonomic function improvements is reached. A key difference is that eSCS is more specific. In contrast, tSCS has a broader effect on tissues, meaning it is less specific in selecting targets within the nervous system.

Taken together, results from eSCS and tSCS show that, in addition to improvements in motor and sensory systems, activating spinal circuits with electrical pulses affects various structures involved in controlling ANS functions. Therefore, it is pertinent to weigh both strategies, emphasizing their similarities and discrepancies, and discussing the limitations identified in the reviewed studies. This narrative review describes how SCI disrupts autonomic function and provides an overview of eSCS and tSCS, detailing the outcomes of these neuromodulation approaches.

### 1.1. Invasive and Non-Invasive Neuromodulation in Spinal Cord Injury: eSCS and tSCS

eSCS has emerged as a promising therapeutic strategy for addressing motor, sensory, and autonomic impairments resulting from SCI. This procedure requires surgically implanting a paddle or multi-electrode array in the epidural space, depending on the intended neural target and study aims, either over the lower cervical, thoracic, or thoraco-lumbar segments.

Depending on the manufacturer, the multi-electrode array may feature up to 60 contact leads. Electrical pulses are delivered below the injury site, intended to enhance residual neural circuits. The electrodes are connected to a pulse generator implanted subcutaneously in the abdominal or gluteal region [14,26,27,28]. eSCS therapeutic potential extends to individuals with complete SCI, as defined by the classification criteria of the American Spinal Injury Association (ASIA) [29]. During the postoperative phase, stimulation parameters such as current intensity or voltage, pulse width, anode/cathode configuration, and the electrode array are adjusted based on the motor-evoked responses observed in the upper or lower limbs during implantation surgery [30]. Notably, voluntary motor responses have been shown in the following days in complete SCI [16,31,32], highlighting the use of eSCS for motor improvement. In addition, an intensive physical rehabilitation program combined with eSCS has been shown to maximize treatment-related movement progress [33,34,35,36].

tSCS is a noninvasive technique that delivers electrical current via adhesive electrodes placed on the skin over intervertebral segments. The most common electrode placement sites are the cervical (C5–7) and thoracolumbar (T11–L1) levels, although researchers have explored other locations as complements, depending on the neural system targeted. Like eSCS, the primary objective of tSCS is to activate residual spinal circuits, thereby enhancing neural activity linked to voluntary functions compromised by SCI. Recent studies have demonstrated that tSCS can promote partial recovery of voluntary movements [37,38,39,40] and modulate autonomic functions in patients with SCI [41,42]. Several factors determine stimulation intensity, including the patient’s tolerance, the presence of involuntary muscle contractions, and the assessment of evoked motor responses [38].

### 1.2. Impact of eSCS and tSCS on Cardiovascular Function in Patients with SCI

Individuals with SCI often experience acute and chronic hemodynamic disturbances, such as hypotension, bradycardia, and hypothermia, due to disrupted supraspinal vasomotor control and autonomic dysfunction. The condition begins immediately post-injury with excessive norepinephrine release, causing reflex-mediated bradycardia, followed by a decline in sympathetic and cardiac activity. Bradycardia often develops within the first 2–3 weeks post-injury due to the loss of supraspinal control over the sympathetic nervous system [43]. This condition is generally less concerning in cases of thoracic SCI, as cardiac sympathetic neurons are regulated by the brainstem [44]. Hypotension occurs in both acute and chronic phases due to reduced sympathetic activity and loss of vasomotor tone. Consequently, individuals experience excessive blood pooling in the lower extremities, along with loss of venous-mitigating muscle function and cardiovascular deconditioning, primarily due to reduced mobility [45].

#### eSCS

With an implanted eSCS device, stimulation parameters such as electrode placement and activation duration determine the resulting cardiovascular effects. As highlighted by the study of Peña Pino et al., safety associated with the device use has also been a matter of concern [46]. In that study, autonomic dysregulation was defined as a sustained increase in blood pressure (BP) of ≥150 mmHg for at least 30 s during a head-up tilt test. Using this criterion, no surgery-related adverse events were reported among the 14 participants, and only one device-related complication occurred, consisting of insufficient battery capacity, during the four months following implantation, in addition to other events unrelated to the surgical procedure. Only two participants exceeded the BP threshold during testing; however, not all participants exhibited autonomic impairment prior to implantation. In this longitudinal study, participants implanted with stimulators at the T11–T12 spinal segments received stimulation across a wide range of parameters, including frequencies between 18 and 700 Hz, pulse widths ranging from 100 to 600 μs, and amplitudes from 0.4 to 17 mA. Overall, the device appeared to be safe under these conditions. However, this conclusion is primarily based on systolic blood pressure behavior, which seemed to stabilize near the predefined upper tolerance threshold. Therefore, the extent to which this finding reflects true cardiovascular safety remains uncertain and motivates further investigation.

In another study involving six participants with complete SCI, a 16-electrode array positioned in the midline at L1–L2 enabled controlled stimulation that increased systolic BP from a hypotensive state to 110–120 mm Hg for about 12 weeks without triggering motor activity [47]. Similarly, in a patient with complete SCI at C4, long-term eSCS application (16-electrode array) at the T11–L1 vertebral level showed significant improvements in cardiovascular stability. After 97 sessions, systolic BP remained within the normal range, central systolic pressure increased, and cerebral blood flow velocity improved, as measured by transcranial Doppler ultrasound [48]. In addition, customizing eSCS therapy in four patients with SCI at C4 led to significant improvements in heart rate (HR) variability and baroreceptor function. These patients underwent daily stimulation for two hours over ≈90 days, with beneficial effects observed during and after the intervention. Stimulation parameters, such as electrode placement and frequency, were tailored to each participant, while pulse intensity remained consistent at 450 μA across all cases. These positive effects were associated with a notable rise in BP during episodes of orthostatic stress [49].

Another case that exemplifies the advantages of eSCS personalization entails a patient with complete SCI at C8 using an 8-electrode epidural stimulator implanted between T11 and L1. Stimulation parameters, including polarity, frequency, and pulse width, were selected to maintain systolic BP between 110 and 120 mmHg. The initial frequency was set at 2 Hz with a pulse width of 250 μs, later adjusted to 25 Hz to promote rhythmic locomotor activity. The current amplitude was initially set at 3 mA and gradually increased to 5 mA to stabilize blood pressure, determined by the orthostatic tilt test results. eSCS effectively sustained normotensive systolic blood pressure, not only during active stimulation sessions but also after the intervention period. Moreover, eSCS significantly alleviated episodes of orthostatic hypotension, enhancing the patient’s ability to tolerate postural changes [50].

Some studies have incorporated eSCS “training sessions” to enhance the cardiovascular effects. These daily 2 h sessions improved BP stability and autonomic function, with sustained effects present through 80 training sessions in individuals with complete cervical SCI who were implanted with a 16-electrode epidural stimulator over the L1 to S1 segments [51]. In another study involving four individuals with complete SCI, participants with a 16-electrode epidural stimulator underwent eSCS sessions in combination with actual physical activity, such as standing and stepping exercises, for one hour per day, five days per week. In this report, physical training integrated eSCS with locomotor or supported standing exercises, resulting in significant improvements in oxygen consumption, aerobic capacity, and cardiovascular function. Standing stimulation frequencies typically ranged from 10 to 40 Hz, whereas stepping stimulation frequencies ranged from 25 to 45 Hz [52].

Despite the diversity of lesion sites, eSCS has been shown to stabilize BP in cases of hypotension and orthostatic dysfunction. Aslan et al. evaluated seven participants (one with C5 SCI, grade B; one with C7 SCI, grade B; one with T2 SCI, grade B; and four with T4 SCI, grade A) with a 16-electrode eSCS epidural stimulator. Notably, eSCS mitigated significant BP fluctuations, allowing participants to maintain an upright posture without experiencing adverse symptoms such as dizziness or fatigue [53].

Concomitantly, cardiovascular adaptations associated with eSCS have been described, including an increased left ventricular mass, interventricular septal thickness, aortic root diameter, and left atrial dimension. Ejection fraction also increased significantly, without changes in diastolic or systolic volumes. A notable rise was observed in the velocity-time integral of cardiac output and stroke volume, while HR remained unaffected. Lastly, diastolic BP increased by 4 ± 1.7 mmHg [54].

#### tSCS

As with eSCS, stimulation parameters (frequency, current intensity, pulse width), variability in session numbers, and the limited number of participants in tSCS research protocols pose significant challenges to establishing consensus on the most effective protocol for autonomic modulation with positive effects on HR and BP. During tSCS, a moderate but statistically significant increase in diastolic BP (6 mmHg) and mean arterial pressure (7 mmHg) was observed across three 24 min sessions, each employing biphasic rectangular pulses delivered through two cervical electrodes (C3–C4 and C6–C7) and one thoracic electrode (T10–T11). In this study, tSCS was applied to 7 children with SCI at a frequency of 30 Hz, modulated by a 10 kHz carrier signal. Intensities ranged from 20 to 70 mA for cervical electrodes and 25 to 190 mA for thoracic electrodes. However, the BP values returned to baseline after the intervention [55].

Longer tSCS administration periods have also been explored. In a cross-sectional study involving 5 patients (4 with cervical and 1 with thoracic lesions), tSCS was applied for 1 h using monophasic pulses with a 1 ms width at 30 Hz. Stimulation intensity was set to 10 mA and gradually increased to 70 mA. Remarkably, tSCS eliminated orthostatic hypotension within the first 60 s of stimulation, demonstrating improvement in cardiovascular function [42].

Although short-term effects of tSCS on cardiovascular function are consistent, less information has been reported on cumulative stimulation effects of long-term stimulation. In this context, longitudinal studies have also shown cardiovascular regulation stability after intense physical activity involving verticalization. Interestingly, current intensity necessary to achieve assisted verticalization with tSCS decreased, as the number of sessions increased, in 15 participants with complete SCI, most with thoracic-level injuries, at a maximum stimulation intensity of 150 mA, with electrode placement at the T11–T12 vertebrae (cathodes) and the iliac crests (anodes). The stimulation protocol consisted of 12 sessions over four weeks, with 120 min sessions conducted three times per week. Monophasic pulses with a 1 ms width were applied at frequencies ranging from 0.2 to 30 Hz, with the optimal frequency determined by motor performance. Notably, no episodes of autonomic dysreflexia were observed with this tSCS protocol [56].

To assess the duration of post-stimulation effects with tSCS, Gad et al., [57] divided the examination into three phases in a case study. The cathodal electrodes were placed between the C3–C4, C5–C6, or T1–T2 vertebrae and the anodal electrode over the bilateral shoulders. During the first phase, an initial one-week evaluation was performed. Phase two consisted of two weeks of stimulation with a high-frequency (10 kHz) biphasic carrier pulse combined with a low-frequency (30 Hz) burst pulse. BP and HR were continuously monitored using portable home-based systems. During the third phase, participants were scheduled for a one-week post-stimulation follow-up. During the tSCS phase, BP increased. However, BP values in the third phase were comparable to those in the first phase, suggesting that tSCS did not provoke persistent changes in cardiovascular function beyond the 1-week intervention.

Improvement of systolic and diastolic BP and mean arterial pressure (MAP) was also observed in two participants with complete thoracic SCI who received tSCS in a first phase before using an eSCS implant. tSCS was delivered as monophasic pulses at frequencies ranging from 0.2 to 30 Hz, modulated by a 10 kHz carrier frequency. Stimulation was administered in a three-hour-per-session protocol, twice per week, over eight weeks, at the maximum tolerable intensity (up to 110 mA). During the initial stimulation phase, both tSCS and eSCS caused episodic increases in systolic BP, reaching 160–180 mmHg in participant 1 (baseline BP: 128/79 mmHg) and 140–160 mmHg in participant 2 (baseline BP: 135/80 mmHg). A few minutes after stimulation, BP returned to normal values, and after 2–3 days of continued stimulation, both patients had BP values in the normal range, regardless of invasive or non-invasive stimulation [58].

While previous results have reported beneficial effects in subjects with cardiovascular dysfunction, a recent study evaluating the presence of autonomic dysreflexia using various tests showed increased sympathetic activity in subjects with SCI. In this study, tSCS was applied at the T10–T11 level at 120 or 30 Hz, with a 5 kHz carrier frequency. The main conclusion was that tSCS did not improve overall autonomic function but instead amplified sympathoexcitatory responses, which could exacerbate dysreflexia events in subjects [59]. Therefore, it is essential to thoroughly explore the mechanisms of tSCS on sympathetic autonomic responses through comprehensive testing that demonstrates beneficial cardiovascular effects without undesirable side effects. In addition, several studies have reported improvements in HR regulation once participants reported a reduction in dizziness. These observations emerged as secondary findings during routine monitoring in training sessions focused on upper-limb motor function recovery rather than as prespecified study outcomes. Consequently, the available evidence remains insufficient to draw definitive conclusions regarding the effects of tSCS on cardiovascular regulation [60].

In summary, both tSCS and eSCS raise BP to physiological levels and prevent orthostatic hypotension. This effect suggests that BP may serve as a useful parameter for informing the selection of personalized stimulation frequency and intensity. However, HR outcomes varied across the cited studies, with some cases showing an increase and others remaining unchanged. The most notable differences between the two techniques are stimulation intensity, which is much lower in eSCS, and the number of sessions, which is considerably lower in tSCS. Naturally, these discrepancies arise from the technical differences between a non-invasive approach (tSCS) and an implanted method (eSCS). Table 1 summarizes studies using both technologies on cardiovascular function.

### 1.3. Effects of eSCS and tSCS on Respiratory Mechanics Following Spinal Cord Injury

The level of the SCI in the individual influences the extent of respiratory muscle innervation loss, directly affecting the breathing cycle [61]. Consequently, SCI above T12 can cause breathing difficulties or even respiratory failure, often requiring assisted ventilation [62]. Thoracic injuries or impairments in bronchial dilation increase the risk of respiratory failure, provoking ANS imbalances that heighten the risk of bradycardia or cardiac arrest during tracheal suctioning, exacerbated by the loss of supraspinal control of the sympathetic nervous system [43]. In addition, damage to accessory respiratory muscles impairs deep inspiration and forced expiration, promoting atelectasis, particularly in the posterior lung lobes [61]. Overall, thoracic muscle dysfunction impairs the clearance of bronchial and alveolar secretions, leading to airway congestion and increasing the risk of infection. While bronchodilators can aid management, excessive mucus production and secretions retained in the airways continue to pose significant challenges to this population [62].

#### eSCS

Various combinations of positioning and simultaneous electrode activation in eSCS affect the respiratory system, primarily by altering muscle strength. In a case report, eSCS was applied in a participant with spinal SCI at the C5–C6 level (ASIA Impairment Scale grade C). When stimulation was applied independently at T9 (40 V, 53 Hz, 150 μs) and L1 (40 V, 53 Hz, 200 μs), airway pressures reached 90 and 82 cm H_2_O, respectively, while peak expiratory flow rates were 6.4 L/s and 5.0 L/s. Interestingly, combining stimulation at both levels produced a significant increase in airway pressure to 132 cm H_2_O, along with a peak expiratory flow rate of 7.4 L/s. In this study, any combination of two or more electrodes produced higher pressures than single-electrode stimulation; however, the T9 + L1 combination proved most effective in terms of pressure intensity [63]. Subsequently, combinations of lower thoracic (T9, T11) and L1 eSCS, particularly T9 + L1, improved respiratory function as measured by expiratory flow rates in 9 subjects with cervical injury. In this study, eSCS was applied at home with initial preconfigured parameters, voltage intensity (10–40 V), and frequency (2–150 Hz). Stimulation parameters were set to maximal positive airway pressure values. This effect was attributed to the relationship between stimulus intensity and increased muscle strength. Particularly, the peak flow values recorded during the study were comparable to those of an effective cough in healthy individuals, thereby reducing the risk of respiratory complications [64]. Another investigation found that the eSCS parameters also have an influence on respiratory rate. Specifically, the study reported a relationship among stimulation frequency (30, 60, and 90 Hz), electrode location (C2, C3, and C6), and respiratory rate during experiments conducted under anesthesia, provoking the largest rate change when the electrode was located over C3 [65]. In summary, eSCS strengthens expiratory muscles, reducing the reliance on external methods for secretion management and representing a promising approach for implant-mediated assisted ventilation and respiratory support.

#### tSCS

In addition to its effects on motor function, tSCS has been reported to improve respiratory function in SCI individuals. A case study using tSCS at 30 Hz, improved respiratory and cough capacity in a SCI subject only after a few days of receiving the intervention [57]. Another study conducted by Kumru et al., combining tSCS with respiratory muscle training, showed significant improvement in dyspnea, hypophonia, maximal inspiratory pressure, maximal expiratory pressure, and forced vital capacity. The study comprised 5 consecutive sessions of tSCS for 30 min. Electrode placement included cathodes at the C3–C4 and T9–T10 spinal levels and two anodes over the iliac crests. Biphasic rectangular pulses of 1 ms duration were delivered at 30 Hz, with a carrier frequency of 10 kHz and a maximum intensity of 120 mA [66]. Again, promising results were observed; however, the short-term evaluation and the lack of assessment of the long-term effects pose several limitations to come to a conclusion on this matter, highlighting the need for confirmatory studies.

Taken together, although evidence on the effects of tSCS and eSCS on respiratory function remains limited, both approaches have shown the ability to enhance respiratory muscle strength, improving respiratory capacity and airflow. This improvement facilitates more effective airway clearance, thereby reducing the risk of pulmonary complications, which are frequent in individuals with high thoracic/cervical injuries. Table 2 provides a summary of the respiratory function results.

### 1.4. Impact of Spinal Cord Neuromodulation on Autonomic Control of Gastrointestinal and Lower Urinary Tract Function in Spinal Cord Injury

Autonomic regulation of gastrointestinal and sphincter function involves a complex interplay between parasympathetic and sympathetic pathways. The parasympathetic innervation of the gastrointestinal tract originates from the vagus nerve, which arises in the brainstem and extends to the splenic flexure of the colon. Beyond this point, sacral spinal roots S2 and S3 contribute to the pelvic plexus, which innervates the splenic flexure, descending colon, and rectum. The internal anal sphincter also receives parasympathetic input from the sacral spinal cord via the pelvic nerves. This innervation facilitates sphincter relaxation by activating muscarinic receptors via preganglionic cholinergic neurons. In contrast, sympathetic innervation of the gastrointestinal tract originates from preganglionic axons emerging from thoracic spinal roots T6 to T12. The lower rectum and anal canal receive input from the aortic and lumbar splanchnic nerves, which converge to form the hypogastric plexus. The internal anal sphincter is innervated by fibers from the inferior mesenteric ganglion via the hypogastric nerves. Sympathetic tone stimulates the smooth muscle of the internal sphincter, helping maintain continence [67].

In individuals with SCI, esophageal dysfunction may manifest as acid reflux, decreased peristalsis, and chest pain. Gastric function is also impaired, with delayed emptying and reduced motility [68]. Colonic dysfunction can lead to constipation, hemorrhoids, cholelithiasis, or pancreatitis, often resulting from immobility, abnormal colonic contractions, altered anorectal tone and reflexes, or the side effects of medications [69]. Another complication that often arises shortly after the injury is paralytic ileus, which typically resolves within 1 to 2 weeks. However, during this period, it is critically important to withhold oral intake of food and liquids, as doing so may lead to vomiting and aspiration. Gastric decompression is therefore routinely performed. Additionally, paralytic ileus can interfere with diaphragmatic movement, potentially causing respiratory difficulties. Over time, individuals with SCI frequently develop anal incontinence due to reduced tone and loss of voluntary control of the external anal sphincter [43]. This condition is also associated with abnormal rectosigmoid distensibility and the loss of recto-anal reflexes [69].

Regarding micturition, the primary anatomical structures involved in bladder function include the detrusor muscle, the bladder neck muscles, the smooth muscles of the urethra, the striated muscles surrounding the urethra, and the pelvic floor muscles. This process is regulated by both peripheral (sympathetic and parasympathetic) and central innervation; while peripheral innervation modulates the contraction and relaxation of the bladder’s smooth muscle, central innervation governs voluntary control over urination [70].

Individuals with SCI face urinary complications, which can be categorized into two types: hyperactive bladder, typically resulting from lesions above the L1 level, which disrupt communication between the pontine micturition center and the sacral spinal segments, and an areflexic bladder, in which detrusor muscle contraction fails to coordinate with the relaxation of the bladder neck and external sphincter. As a result, these structures remain closed, leading to impaired bladder emptying [71]. As a result, subjects with SCI experience urinary incontinence, and in some cases, residual urine and elevated intravesical pressures.

#### eSCS

Motor and general autonomic improvements with the use of eSCS have been reported; however, proper electrode configuration to provide body system specificity has not been evaluated. Bladder monitoring, high-resolution imaging, and computational modeling are key to improve urinary function monitoring in subjects with complete SCI.

A system-specific protocol included a 16-electrode array implanted in 7 participants, targeted to activate spinal segments, including L3–4 for bladder compliance and L4–S1 for initiating voiding, using stimulation frequencies > 60 Hz. This location and stimulation parameters produced better results compared to the typical 30 Hz used for facilitation of motor responses [72]. The “urge to void” sensation was assessed in a participant receiving eSCS over the T8–T10 spinal segments. Using 120-μs square pulses with stimulation intensities ranging from 1 to 23 mA, the participant consistently reported an immediate urge to void at currents between 14 and 15 mA [66]. These findings suggest that eSCS may provide artificial sensory feedback related to bladder filling, potentially allowing individuals to monitor bladder fullness and initiate voiding at an appropriate time.

To assess bladder and bowel dysfunction, the most commonly used questionnaires in the reviewed literature were the Neurogenic Bladder Symptom Score (NBSS) and the Neurogenic Bowel Dysfunction Score (NBDS). In a study of three female participants with complete SCI who underwent eSCS (16 electrodes, T11–T12), the NBSS changed from 7.0 ± 7.0 pre-intervention to 4.3 ± 7.5 post-intervention, indicating improved function. Similarly, the NBDS showed improvements, decreasing from 12.0 ± 3.6 to 10.7 ± 2.9. Both NBSS and NBDS scores reflected improvements in urinary storage and voiding functions, with notable reductions in incontinence and enhanced voluntary urination. This study did not report specific eSCS parameters [73]. In another study, two female participants with complete SCI (T4 and T8, respectively) and an eSCS device implanted at T12 experienced a reduction in the time required for bowel management routines [32]. These improvements are critical for enhancing the patient’s quality of life and independence; however, they are not explicitly represented by existing standardized scales. Regarding sphincter management, the Digital Rectal Examination Scoring System (DRESS) evaluates functional improvements. In a case report, eSCS was applied in an individual with complete SCI at the L1 level (ASIA grade A) who reported a decrease in bowel incontinence episodes from 4 per week to 3 per month, resulting in a 1-point improvement on the DRESS. Although voluntary anal contraction and deep anal pressure sensation were not recovered, the patient raised 3 points on the Spinal Cord Independence Measure (SCIM). In this study, the eSCS array was implanted from T12 to L1, and the stimulation frequency was set at 40 Hz [74].

#### tSCS

Encouraging results have also been observed in patients treated with non-invasive electrical stimulation. A study by Gad et al. [75], reported improved voiding efficiency, increasing from 26.99 ± 15.41% to 50.80 ± 5.25%, in 7 patients. Moreover, tSCS at 30 Hz enhanced bladder capacity and detrusor-sphincter coordination during voiding, with bladder capacity increasing from 170.54 ± 15.86 mL to 252.59 ± 18.91 mL. Interestingly, low-frequency stimulation (0.5 Hz and 1 Hz), starting at 10 mA and increasing in 10 mA increments to 200 mA, mapped responses and facilitated voiding without inducing sustained contractions. In contrast, 30 Hz stimulation modulated bladder activity to induce a controlled detrusor contraction rather than a spontaneous reflex contraction, suggesting that stimulation parameters play a critical role in targeting specific organ functions [75]. In another study by the same group, urodynamic assessments, including intravesical, urethral sphincter, and abdominal pressures, revealed significant improvements in 5 participants with SCI. These improvements included reduced detrusor overactivity, increased bladder capacity, and enhanced detrusor-sphincter coordination, resulting in more efficient voiding. Furthermore, NBSS decreased significantly, and incontinence episodes were reduced by 68%. In these cases, cathodes were placed over the interspinous ligaments at T11 and L1, with anodes positioned on the iliac crests (biphasic, 30 Hz, 10 kHz carrier signal) [76].

While some studies cited above using tSCS have focused on evaluating bladder function [75,76], the overlap of electrodes in lumbar or cervical segments to enhance motor output and improve autonomic function is interesting [60,77]. For example, combined tSCS (30 Hz) and training aimed to restore upper limb function also showed improvements in urinary function. In a first study, Inanici et al., described a decreased post-void residual urine volume from an initial range of 175–200 mL to 100–125 mL in a 62-year-old male. The protocol consisted of a 9-week tSCS period, with sessions lasting 2 h per day, 4 to 5 days per week, combined with a structured physical rehabilitation program. This reduction led to a notable improvement in bladder-related quality of life, as measured by the SF-Qualiveen questionnaire. In this study, tSCS was applied at 30 Hz with a 10 kHz carrier frequency. The electrical current was delivered in C3–4 and C6–7 [77]. Subsequently, the same group reported improved voluntary voiding control and reduced post-void residual urine in 2 of 6 patients who used intermittent catheterization for bladder management [60].

On voluntary voiding, two participants who had received prior voluntary voiding training were able to initiate voluntary urination exclusively during tSCS. Furthermore, combined thoracic and lumbar stimulation resulted in a greater voided volume compared with stimulation at a single spinal site [65]. Nevertheless, unintentional micturition reflexes may occur during stimulation, as reported in a study in which 3 of 15 participants exhibited this response during standing position and tSCS at T11–L2 [56].

A previously described publication also reported subjective improvements in urinary function (1/2 participants) assessed by the NBSS with tSCS followed by eSCS [58]. Similarly, anecdotal, subjective benefits, including absence of urinary infections and improved bowel management, have been reported [40,78].

As noted, few studies have evaluated the effects of tSCS and eSCS on gastrointestinal and bladder function. However, improvements have been observed in validated scales for urinary and fecal incontinence, intestinal motility, among other clinical outcomes. Overall, the results show benefit in the patients’ independence and quality of life. Importantly, future research should include larger samples and quantitative measures to determine whether this modest amelioration is consistent across patients with different SCI levels and severities. Additionally, developing scales that detect subtle changes in digestive and excretory functions would be valuable, given the significant impact these functions have on the quality of life of individuals with SCI. Table 3 summarizes the main findings of bladder and bowel function using eSCS and tSCS.

### 1.5. Effects of eSCS and tSCS on Other Autonomic Functions

#### 1.5.1. Thermoregulation

Thermoregulatory function is primarily controlled by the sympathetic nervous system through pilomotor, sudomotor, and vasomotor structures. Heat exposure or physical activity triggers vasodilation and sweating to dissipate heat, while cold exposure induces vasoconstriction to conserve heat. In individuals with SCI, disruption of supraspinal sympathetic pathways below the level of injury impairs thermoregulatory responses, making them vulnerable to environmental temperature fluctuations. As a compensatory response, sweat gland activity above the injury site increases. Consequently, the body cannot effectively dissipate heat during fever or in hot environments. As injury severity increases and the area of thermosensitive skin decreases, both afferent and efferent thermoregulatory pathways are further impaired. Therefore, individuals with cervical SCI exhibit more severe thermoregulatory dysfunction compared to those with thoracic injuries [43,45,79].

##### tSCS

In a study of cervical tSCS, two participants improved sweating below the injury level [78]. Similarly, Inanici et al., reported thermoregulatory improvements in three patients with SCI, as reflected by increased SCIM scores following tSCS [60]. In another study, tSCS enhanced cold tolerance in a participant with incomplete SCI at C6 (AIS grade D). Stimulation was applied at spinal levels C3–4, C6–7, T11, and L1 (cathodes), with anodes placed over the iliac crests. The stimulation frequency was set to 30 Hz, with a 10 kHz carrier frequency. This configuration allowed the patient to remain in a cold environment longer without discomfort [40]. These results were reported subjectively and as secondary outcomes, thus being preliminary, highlighting the need for systematic evaluation in future studies. Regarding eSCS, our literature review found no studies reporting its effects on thermoregulatory function.

#### 1.5.2. Sexual Dysfunction

Sexual function is regulated by autonomic innervation of the genitalia, primarily through thoracolumbar and sacral spinal segments. Parasympathetic activation and sympathetic inhibition are essential for reflex sexual arousal, leading to vasodilation. Orgasm is mediated by a spinal reflex. In men, this includes penile erection, ejaculation, and rhythmic contractions of the perineal muscles; in women, vaginal and uterine contractions occur alongside similar perineal muscle activity. In SCI, the disruption of communication between the brain and spinal cord impairs sexual responses. However, in individuals with SCI, sexual arousal may be predominantly mediated by the sympathetic nervous system [45]. Men with SCI often experience erectile dysfunction, ejaculatory disorders, and altered orgasmic sensation, which can significantly affect self-esteem and intimate relationships. Although 95% of men with SCI may still experience erections, these are typically insufficient in rigidity and duration for penetrative intercourse. The loss of coordination between erectile and ejaculatory reflexes further impairs sexual function. Psychogenic ejaculation is generally preserved in injuries below T10, while it is absent in injuries above this level [80]. Sexual dysfunction in women with SCI commonly includes reduced vaginal lubrication, diminished sexual desire and arousal, and difficulties achieving orgasm. Additionally, involuntary urinary leakage during sexual activity can significantly impact sexual well-being, leading to feelings of embarrassment, reduced self-esteem, and a marked decline in overall sexual quality of life [81,82].

##### eSCS

To evaluate improvements in sexual function, validated scales have been employed to assess the effects of spinal cord electrical stimulation. In one eSCS study, three women with SCI (injuries at T4, T5, and T8) received continuous electrical stimulation for 24 h per day for 13 months. Monthly assessments included the Female Sexual Function Index and the Female Sexual Distress Scale. Results showed an average increase of 3.2 points (13.2%) in the total FSFI score, with improvements ranging from 4.8% to 50% across the desire, arousal, orgasm, and satisfaction subdomains. Sexual distress decreased by 55%, with a mean reduction of 12 points. Additionally, the total sensory score on the International Standards for Neurological Classification of Spinal Cord Injury improved by a clinically meaningful 14 points, increasing from 102 ± 10.5 at baseline to 116 ± 17.4 [73]. Other validated instruments include the International Index of Erectile Function-5 (IIEF-5), which assesses erectile function, and the Quality of Life Brief questionnaire (WHOQOL-BREF), which evaluates satisfaction with personal well-being and social circumstances. Rybka et al., conducted an eSCS study in two male patients with complete SCI (T4 and T5, AIS grade A) who were implanted with a 32-electrode epidural stimulator targeting spinal segments L1–S1. Three months post-intervention, the IIEF-5 score improved from 7 to 14 points, while the WHOQOL-BREF score increased from 15.1 to 16.0, indicating enhanced sexual function and perceived quality of life [83].

The ability to achieve orgasm has also been reported with eSCS (16-electrode array implanted at T12) during sexual intercourse, both while the stimulation was active and immediately afterward, although precise stimulation frequency was not mentioned; a range between 16 and 400 Hz was adjusted to optimize the desired responses on motor and autonomic functions [32].

##### tSCS

We found only one study in which 1/8 participants (AIS grade B) reported increased duration of reflex erections with tSCS delivered at C3–C4 and C6–C7 (30 Hz, 90–140 mA), although the study intended to improve upper-limb function [78].

Despite a general trend toward improved scores on scales assessing thermoregulatory and sexual function following tSCS and eSCS, the heterogeneity of assessment tools and small samples limit the ability to draw definitive conclusions about their efficacy; therefore, results must be taken cautiously and considered exploratory. Additionally, most of the studies reviewed do not specifically focus on stimulation-induced changes in autonomic function. We therefore suggest that future research incorporate dedicated instrumentation to quantitatively assess autonomic parameters and systematically evaluate the effects of neuromodulation on autonomic function. Such approaches would enhance the interpretability of whether electrical stimulation yields clinically meaningful benefits in autonomic domains. Table 4 summarizes the results of this section.

Table 5 summarizes the clinical characteristics of subjects enrolled in this narrative review. Most included studies included more males than females, although inclusion criteria usually included both sexes. This may reflect the higher SCI prevalence reported in males [84]. Future studies may systematically evaluate whether outcomes vary between sexes. Also, studies specifically designed to systematically evaluate sexual function are desirable, as part of the health priorities for injured subjects [85]. Furthermore, the wide ranges in both time since injury and participants’ ages make it difficult to determine whether these factors decisively influence outcomes. Additionally, the severity of the spinal cord injury is another variable that adds to the overall picture. Therefore, differences in participants’ baseline clinical profiles substantially influence autonomic responses to neuromodulation.

## 2. Conclusions

**Figure 1:**
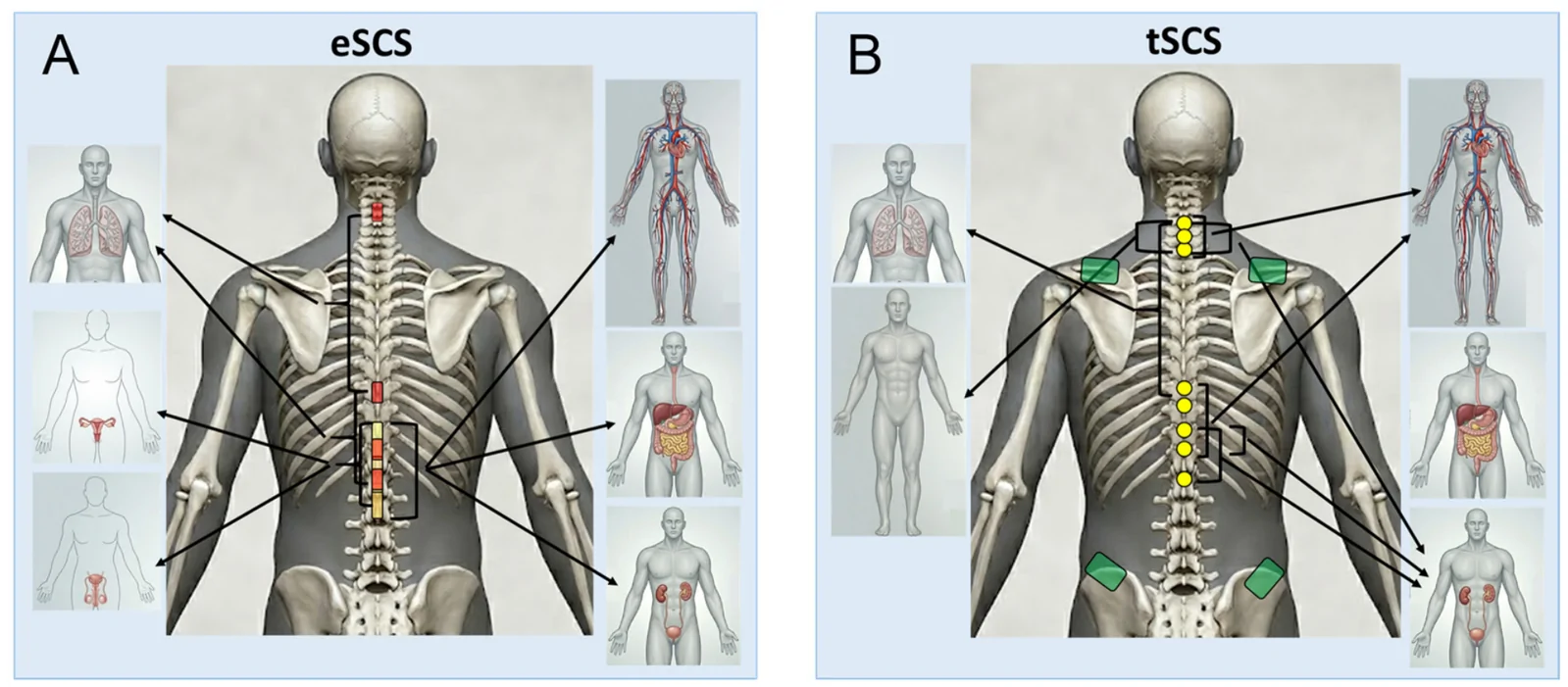
(**A**) Invasive eSCS placement of multielectrode arrays and targeted body systems. Red circles at the cervical level represent stimulation at single spinal cord segments, while rectangles span the multielectrode arrays common locations s across thoracic and lumbar segments. (**B**) Non-invasive placement of electrodes aimed at delivering tSCS to targeted body systems. Circles represent cathodes in intervertebral locations, and green rectangles represent the anodes. Note: electrode locations for both eSCS and tSCS are representative of configurations reported; they do not represent standardized or recommended clinical placements.

This review provides a general overview of ANS pathophysiology following SCI. Given the current lack of clinical treatments to mitigate the consequences of impaired autonomic neural control following SCI, the study of new strategies is highly relevant. In particular, neuromodulation using both eSCS and tSCS has shown promising results. Both approaches aim to improve motor function recovery in individuals with SCI; however, these neuromodulation techniques not only improve muscle tone and voluntary mobility but also positively influence other physiological systems, including cardiovascular-respiratory, gastrointestinal-excretory, thermoregulatory, and sexual functions. Although electrode placement over the cervical and thoracolumbar enlargements of the spinal cord has been explored most frequently, other locations have been investigated in a complementary manner. Figure 1 summarizes the placement of eSCS multielectrode arrays and tSCS electrodes (Figure 1A,B, respectively), as well as the body systems targeted by each approach.

The most extensive evidence has evaluated HR and BP, while few studies have addressed other autonomic functions such as thermoregulation and sexual function. Overall, improvements in certain clinical parameters have been observed across the systems evaluated; however, benefits appear transient, as improvements tend to return to baseline once stimulation is discontinued. Although improvements on clinical scales may not be remarkable, patient-reported quality-of-life and personal-independence outcomes underscore the significant impact of these modest gains on daily living. In this context, developing or adopting more sensitive clinical scales and objective measures would help capture the subtle yet meaningful changes induced by electrical stimulation.

A significant limitation to translating these neuromodulation strategies to clinical practice stems in part from sample size, with most reports being case studies and case series. However, results across SCI-affected areas have encouraged protocols with larger sample sizes and blinding, which will likely yield more robust data than is currently available. Overall, we encountered several challenges in synthesizing findings on autonomic functions, primarily because evaluation parameters and scales varied across studies and autonomic outcomes were often secondary to primary assessments of motor function. Finally, we emphasize the importance of incorporating biomedical instrumentation specifically designed to evaluate physiological phenomena, particularly in gastrointestinal-excretory, thermoregulatory, and sexual domains, to complement data obtained from clinical and quality-of-life scales. While we acknowledge the technical challenges and potential patient discomfort, these instruments suggest that the cost–benefit ratio of integrating physiological measurements could enable more precise characterization of the autonomic changes induced by both tSCS and eSCS in individuals with SCI.

In this narrative review, we presented findings on both eSCS and tSCS, grouped by autonomic systems; however, we did not aim to compare their effectiveness directly. Consequently, given the heterogeneity of the literature and variations in primary and secondary objectives and anecdotal results, we cannot conclude superiority or equivalence between these neuromodulation interventions. Additionally, we acknowledge that the lack of a systematic literature search methodology, potential bias in article selection, and the specific criteria for selecting results are limitations inherent to a narrative review.

Finally, although most articles report no major adverse effects, neither eSCS nor tSCS is free of risk for participants. For instance, with eSCS, the multi-electrode array could shift and potentially cause tissue damage or malfunction, in addition to the risks inherent in any surgical procedure, such as infection. Regarding tSCS, a key factor to monitor is contact between the stimulation electrodes and the skin; applying electric current and changes in tissue impedance can potentially cause skin damage. Furthermore, while most studies included in this narrative review assess the effects that could mitigate orthostatic hypotension and improve cardiovascular performance, some suggest that tSCS might exacerbate episodes of dysreflexia [59]. Given this, and considering heterogeneity in stimulation patterns and sites for both neuromodulation strategies, as well as the diversity of study populations, it is essential to evaluate the safety of both eSCS and tSCS before clinical translation to ensure the benefits outweigh the potential risks.

## Figures and Tables

**Table 1:** Summary of eSCS and tSCS studies on cardiovascular function.

Author(s)	eSCS or tSCS	# of Participants	Study Design	Electrode Location	Main Objective	Main Outcome(s) in Cardiovascular Function
Angeli et al., 2025 [36]	eSCS	30	Cross sectional	L3–L4	Motor and autonomic function (Primary)	Stabilize blood pressure (Secondary)
Darrow et al., 2019 [32]	eSCS	2	Cases report	L1–S2	Motor and autonomic function (Primary)	Cardiovascular function restored during tilt challenge in 1/2 subject (Primary)
Legg Ditterline et al., 2020 [47]	eSCS	6	Longitudinal quasi-experimental	L1–L2	Impact on lower limb muscle properties (Primary)	Parameters optimized for cardiovascular function normalization promote lower limb lean mass augmentation (Primary)
Bloom et al., 2020 [48]	eSCS	1	Case report	L1–S1	Improved orthostatic tolerance (Primary)	Normalization of blood pressure parameters promoted positive immunological changes (Secondary)
Legg Ditterline et al., 2021 [49]	eSCS	4	Case series	L1–S1	Cardiovascular function (Primary)	Sustained increase in HR variability and baroreceptor improvement before and during orthostatic stress. (Primary)
Gorgey et al., 2023 [50]	eSCS	1	Case report	T11–L1	Cardiovascular function and spasticity (Primary)	Beneficial effects on cardiovascular function (Primary)
Harkema et al., 2018 [51]	eSCS	4	Early feasibility study	L1–S1	Cardiovascular function (Primary)	Alleviation of orthostatic hypotension (Primary)
Terson de Paleville et al., 2019 [52]	eSCS	4	Case series	L1–S1	Metabolic composition and cardiovascular fitness (Primary)	Improvements of cardiovascular function and body composition (Secondary)
Aslan et al., 2018 [53]	eSCS	7	Case series	L1–S1	Arterial blood pressure (Primary)	Increased arterial blood pressure in individuals with SCI-induced cardiovascular impairment (Primary)
Pino et al., 2022 [46]	eSCS	14	Cohort longitudinal prospective study	Twelfth thoracic vertebra level	Post-surgical complications and cardiovascular events (Primary)	No post-operative related complications. Two subjects experienced elevated systolic blood pressure during tilt-table test. (Primary)
Legg Ditterline et al., 2020 [54]	eSCS	4	Pilot study	L1–S1	Structural and Functional Modifications of the Heart (Primary)	Task-oriented interventions and eSCS resulted in positive cardiac remodeling (Primary)
Singh et al., 2023 [55]	tSCS	7	Proof-of-principle	C3–4, C6–7 and T10–11	Upper extremity strength (Primary)	Hemodynamic parameters remained stable through the assessment (Secondary)
Phillips et al., 2018 [42]	tSCS	5	Case series	Between TVII–TVIII (T8 spinal segment)	Cardiovascular function (Primary)	Blood pressure normalization, cardiac contractility and cerebral flow improvement (Primary)
Sayenko et al., 2019 [56]	tSCS	15	Double-blind, crossover, balanced, sham-controlled	T11–12	Self-assisted standing (Primary)	No episodes or autonomic dysreflexia were observed (Secondary)
Gad et al., 2020 [57]	tSCS	1	Exploratory, case study	C3–C4, C5–C6, or T1–T2	Respiratory control (Primary)	Increased BP, although tSCS did not provoke persistent changes in cardiovascular function beyond a 1-week intervention. (Secondary)
Mukhametova et al., 2024 [58]	tSCS + eSCS	2	Proof-of- concept	Th11–12 and Th12–L1 (cathode) low abdomen (anode)/Dorsal epidural lumbosacral	Volitional movements (Primary)	BP increased and normalized after 20 min, maintaining normal ranges (Secondary)
Solinsky et al., 2023 [59]	tSCS	4; 2 w SCI 2 w/o SCI	Head-to-head study	T–10, T11 (cathode), bilateral iliac crests (anode)	Cardiovascular regulation (Primary)	Exacerbated impaired sympathoexcitatory response in provocative autonomic maneuvers (Primary)
Inanici et al., 2021 [60]	tSCS	6	Cross-over study	Neck midline, one above and one below injury (cathode), iliac crests (anode)	Hand and Arm function (Primary)	Heart rate normalization in one subject. (Secondary)

w with; w/o without. Note: “Primary” refers to the main result reported by the authors, whereas “Secondary” refers to complementary results that are not the study’s main objective. The study design is presented as originally described in the primary publication. # Number.

**Table 2:** Summary of eSCS and tSCS studies on respiratory function.

Author(s)	eSCS or tSCS	# of Participants	Study Design	Electrode Location	Main Objective	Main Outcome(s) In Respiratory Function
Rezaii, 2016 [65]	eSCS	1	Case report	C3–C4	Respiratory function (Primary)	Respiratory rate and tidal volume modulated by stim parameters (Primary)
DiMarco et al., 2006 [63]	eSCS	1	Case report	T9, T11 and L1	Restore cough (Primary)	Effective throat clearance, quality of life improvement (Primary)
DiMarco et al., 2009 [64]	eSCS	9	Clinical trial	T9, T11 and L1	Restore cough (Primary)	Secretion clearance improvement, incidence reduction in acute respiratory tract infections. (Primary)
Gad et al., 2020 [57]	tSCS	1	Exploratory, case study	Cathode: C3–C4, C5–C6 or T1–T2 Anode: bilateral shoulders	Respiratory control (Primary)	Positive effects on breathing and coughing immediately and days after stimulation. (Primary)
Kumru et al., 2023 [66]	tSCS	11	Randomized control trial	C3–C4 and Th9–Th10 (cathodes), iliac crests (anode)	Respiratory muscles strength (Primary)	Significant improvement in dyspnea, hypophonia, forced vital capacity, and maximal inspiratory/expiratory pressure (Primary)

Note: “Primary” refers to the main result reported by the authors, whereas “Secondary” refers to complementary results that are not the study’s main objective. The study design is presented as originally described in the primary publication. # Number.

**Table 3:** Summary of eSCS and tSCS studies on bladder and bowel function.

Author(s)	eSCS or tSCS	# of Participants	Study Design	Electrode Location	Main Objective	Main Outcome(s) in Bladder and Bowel Function
Rezaii, 2016 [65]	eSCS	2	Case report	T8–T10	Bladder function (Primary)	Participant indicated urge state before voiding (Primary)
Herrity et al., 2022 [72]	eSCS	7	Pilot clinical trial	T11–L1	Bladder function (Primary)	Modulation of urinary continence and initiation of voiding (Primary)
Shackleton et al., 2023 [73]	eSCS	3	Single-blinded, randomized sham-controlled trial	T11–12	Female sexual function (Primary)	Subjective improvements in urinary function (NBSS) (Secondary)
Darrow et al., 2019 [32]	eSCS	2	Cases report	L1–S2	Motor and autonomic function (Primary)	Bowel-bladder synergy restoration (Primary)
Krucoff et al., 2020 [74]	eSCS	1	Case report	T12–L1 epidural space	Motor and functional outcomes (Primary)	Improved anal tone, bowel incontinence, and bladder fullness sensation (Secondary)
Gad et al., 2018 [75]	tSCS	7	Pilot study	T11–T12 and L1–L2 (cathode), iliac crests (anode)	Urinary function (Primary)	Decreased detrusor overactivity, detrusor-sphincter dyssynergia, increased bladder capacity and voiding. (Primary)
Kreydin et al., 2020 [76]	tSCS	14 (SCI = 5)	Pilot study	T11 and L1 cathode, iliac crests (anode)	Bladder function (Primary)	Reduction in detrusor overactivity, incontinence episodes, increased bladder capacity, and bladder fullness sensation (Primary)
Inanici et al., 2018 [77]	tSCS	1	Case study	C3–C4, C6–C7 (cathode), iliac crests (anode)	Recovery of upper extremity function (Primary)	Decreased residual volume (Secondary)
Inanici et al., 2021 [60]	tSCS	6	Cross-over study	Neck midline, one above and one below injury (cathode), iliac crests (anode)	Hand and Arm function (Primary)	Improved volitional voiding and decreased residual volume in 2 subjects (Secondary)
Mukhametova et al., 2024 [58]	tSCS + eSCS	2	Proof-of- concept	Th11–12 and Th12-L1 (cathode) low abdomen (anode)/Dorsal epidural lumbosacral	Volitional movements (Primary)	Bladder fullness sensation, improved bladder control, and induced urination (Secondary)
Samejima et al., 2022 [40]	tSCS	2	Single-subjects	C3–4, C6–7, T11 and L1 (active), iliac crests (ground)	Walking and autonomic (Primary)	Bowel function improvement (Secondary)
Gad et al., 2018 [78]	tSCS	6	Interventional	C3–C4, C6–C7 (cathode), iliac crests (anode)	Voluntary control of hand (Primary)	Increased bladder and bowel control. Increased bladder fullness sensation (Secondary)

Note: “Primary” refers to the main result reported by the authors, whereas “secondary” refers to complementary results that are not the study’s main objective. The study design is presented as originally described in the primary publication. # Number.

**Table 4:** Summary of eSCS and tSCS studies on sexual function.

Author(s)	eSCS or tSCS	# of Participants	Study Design	Electrode Location	Main Objective	Main Outcome(s) in Sexual Function
Shackleton et al., 2023 [73]	eSCS	3	Single-blinded, randomized sham-controlled trial	T11–T12 vertebral level	Female sexual function (Primary)	Increment on Female Sexual Function Index and decrement on Female Sexual Distress Scale (Primary)
Rybka et al., 2024 [83]	eSCS	2	Cases report	L1–S1	Ejaculatory response (Primary)	Restoration of ejaculatory reflex (Primary)
Darrow et al., 2019 [32]	eSCS	2	Cases report	L1–S2	Motor and autonomic function (Primary)	Orgasm restoration in 1 subject (Secondary)
Krucoff et al., 2020 [74]	eSCS	1	Case report	T12–L1 epidural space	Motor and functional outcomes (Primary)	Subjective erection and ejaculation control improvement I/A (Secondary)
Gad et al., 2018 [78]	tSCS	8	Interventional	C3–C4, C6–C7 (cathode), iliac crests (anode)	Voluntary control of hand (Primary)	Regained sexual function I/A (Secondary)

I/A = Incidental/Anecdotal. Note: “Primary” refers to the main result reported by the authors, whereas “secondary” refers to complementary results that are not the study’s main objective. # Number.

**Table 5:** Summary of clinical characteristics of subjects enrolled in eSCS and tSCS studies.

Author(s)	eSCS or tSCS	NL	AIS	Sex F/M	Time from Injury (Years)	Age of Participants *
Angeli et al., 2025 [36]	eSCS	C3-1, C4-21 C5-4, C6-2 C7-1, C8-1	A-16, B-12, C-2	16 F 14 M	2.4–38.6	20–59
Darrow et al., 2019 [32]	eSCS	T4-1, T8-1	A-2	2 F	5, 10	48, 52
Legg Ditterline et al., 2020 [47]	eSCS	C4-4, C5-1, C6-1	A-2, B-4	1 F 5 M	3.8–10	24–43.3
Bloom et al., 2020 [48]	eSCS	C4	A	1 F	3.1	27
Legg Ditterline et al., 2021 [49]	eSCS	C4-4	A-3, B-1	1 F 3 M	3.8–8.0	24–35
Gorgey et al., 2023 [50]	eSCS	C8	A	1 M	7	27
Harkema et al., 2018 [51]	eSCS	C4-4	A-3, B-1	1 F 3 M	3.8–8.0	24–35
Terson de Paleville et al., 2019 [52]	eSCS	C5-1, T4-2 T5-1	A-3, B-1	4 M	2.3–2.9	22.7–31.6
Aslan et al., 2018 [53]	eSCS	C5-1, C7-1 T2-1, T4-4	A-4, B-3	7 M	2.0–3.5	26.7 ± 4.1
Pino et al., 2022 [46]	eSCS	T3-1, T4-6, T5-2, T6-1, T8-3, T10-1	A-11, B-3	3 F 11 M	1.6–16.8	26–58
Legg Ditterline et al., 2020 [54]	eSCS	C4-2, C5-1, C6-1	A-3, B-1	1 F 3 M	3.8–7.9	21–31
Singh et al., 2024 [55]	tSCS	C1-1, C2-1 C3-1, C4-1, C5-1, C6-1, C7-1	A-1, B-4, C-1, D-1	1 F 6 M	2–5	6–17
Phillips et al., 2018 [42]	tSCS	C5-3, C6-1, T2-1	A-3, B-2	1 F 4 M	>3	23–32
Sayenko et al., 2019 [56]	tSCS	C4-1, C5-3, C6-1, T1-1, T2-2, T3-2, T4-2, T8-1, T9-2	A-11, B-1 C-3	3 F 12 M	2–13	23–53
Gad et al., 2020 [57]	tSCS	C5	A	1 M	9	39
Mukhametova et al., 2024 [58]	tSCS + eSCS	T4-1, T5-1	A-1, B-1	1 F 1 M	2, 5	28, 48
Solinsky et al., 2023 [59]	tSCS	T3-1, T5-1	A-2	1-M 1-F (vs. two well-matched pairs)	NA	20–30
Inanici et al., 2021 [60]	tSCS	C3-1, C5-5	B-2, C-2, D-2	2 F 4 M	1.5–12	28–62
Rezaii, 2016 [65]	eSCS	T3-1, T3/T4-1	A-1, B-1	2 M	NA	45–58
Di Marco et al., 2006 [63]	eSCS	C5-C6	C	1 M	7	52
Di Marco et al., 2009 [64]	eSCS	C3-2, C3/C4-1, C4-1, C4/C5-2, C5/C6-3	NA	1 F 8 M	1–34	23–52
Kumru et al., 2023 [66]	tSCS	C3-1, C4-5, C5-2, C6-1, C7-2	A-2, B-4, C-4, D-1	1 F 10 M	<1	29.3 ± 10.1
Herrity et al., 2022 [72]	eSCS	C3-1, C4-2, C5-1, C7-1, C8-1, T2-1	A-2, B-5	1 F 6 M	5–13	26–39
Shackleton et al., 2023 [73]	eSCS	T4-1, T5-1, T8-1	A-3	3 F	11.7 ± 3.1	47.7 ± 11.2
Krucoff et al., 2020 [74]	eSCS	L1	A	1 M	7	48
Gad et al., 2018 [75]	tSCS	C3-1, C6-1, T4-1, T6-2, T8-1, T11-1	A-4, B-1, C-2	3 F 4 M	1–23	28–49
Kreydin et al., 2020 [76]	tSCS	C4-1, T4-1, T5-1, T6-1, T9-1	A-3,C-2	1 F 4 M	1–11	25–60
Inanici et al., 2018 [77]	tSCS	C3	D	1 M	2	62
Samejima et al., 2022 [40]	tSCS	C4-1 C-6	D-2	2 M	3.5–4.5	64
Rybka et al., 2024 [83]	eSCS	T4-2	A-2	2 M	2, 3.5	25, 32
Gad et al., 2018 [78]	tSCS	C4-3, C6-2, C8-1	B-2, C-4	1 F 5 M	1–21	20–62

NL, Neurological level; AIS, ASIA Impairment Scale; NA, Not Available on original source; * reported mean and standard deviation (mean ± SD) or range according to the original source.

## Data Availability

All analyzed data are already cited within the article.

## Abbreviations

The following abbreviations are used in this manuscript:

SCI	Spinal Cord Injury
AD	Autonomic Dysfunction
eSCS	Epidural Spinal Cord Stimulation
tSCS	Transcutaneous Spinal Cord Stimulation
ANS	Autonomic Nervous System
ASIA	American Spinal Injury Association
BP	Blood pressure
HR	Heart rate
NBSS	Neurogenic Bladder Symptom Score
NBDS	Neurogenic Bowel Dysfunction Score
DRESS	Digital Rectal Examination Scoring System
SCIM	Spinal Cord Independence Measure
IIEF-5	International Index of Erectile Function-5
WHOQOL-BREF	World Health Organization Quality of Life Brief version
